# Oral Ondansetron Administration to Nondehydrated Children With Diarrhea and Associated Vomiting in Emergency Departments in Pakistan: A Randomized Controlled Trial

**DOI:** 10.1016/j.annemergmed.2018.09.011

**Published:** 2019-03

**Authors:** Stephen B. Freedman, Sajid B. Soofi, Andrew R. Willan, Sarah Williamson-Urquhart, Noshad Ali, Jianling Xie, Fady Dawoud, Zulfiqar A. Bhutta

**Affiliations:** a Section of Pediatric Emergency Medicine, Cumming School of Medicine, University of Calgary, Calgary, Alberta, Canada; b Section of Pediatric Gastroenterology, Cumming School of Medicine, University of Calgary, Calgary, Alberta, Canada; c Department of Paediatrics, Alberta Children’s Hospital, Alberta Children’s Hospital Research Institute, Cumming School of Medicine, University of Calgary, Calgary, Alberta, Canada; d Centre of Excellence in Women and Child Health, Aga Khan University, Karachi, Pakistan; e Ontario Child Health Support Unit, Sickkids Research Institute, Toronto, Ontario, Canada; f Centre for Global Child Health, The Hospital for Sick Children, Toronto, Ontario, Canada

## Abstract

**Study objective:**

We determine whether single-dose oral ondansetron administration to children with vomiting as a result of acute gastroenteritis without dehydration reduces administration of intravenous fluid rehydration.

**Methods:**

In this 2-hospital, double-blind, placebo-controlled, emergency department–based, randomized trial conducted in Karachi Pakistan, we recruited children aged 0.5 to 5.0 years, without dehydration, who had diarrhea and greater than or equal to 1 episode of vomiting within 4 hours of arrival. Patients were randomly assigned (1:1), through an Internet-based randomization service using a stratified variable-block randomization scheme, to single-dose oral ondansetron or placebo. The primary endpoint was intravenous rehydration (administration of ≥20 mL/kg of an isotonic fluid during 4 hours) within 72 hours of randomization.

**Results:**

Participant median age was 15 months (interquartile range 10 to 26) and 59.4% (372/626) were male patients. Intravenous rehydration use was 12.1% (38/314) and 11.9% (37/312) in the placebo and ondansetron groups, respectively (odds ratio 0.98; 95% confidence interval [CI] 0.60 to 1.61; difference 0.2%; 95% CI of the difference –4.9% to 5.4%). Bolus fluid administration occurred within 72 hours of randomization in 10.8% (34/314) and 10.3% (27/312) of children administered placebo and ondansetron, respectively (odds ratio 0.95; 95% CI 0.56 to 1.59). A multivariable regression model fitted with treatment group and adjusted for antiemetic administration, antibiotics, zinc prerandomization, and vomiting frequency prerandomization yielded similar results (odds ratio 0.91; 95% CI 0.55 to 1.53). There was no interaction between treatment group and age, greater than or equal to 3 stools in the preceding 24 hours, or greater than or equal to 3 vomiting episodes in the preceding 24 hours.

**Conclusion:**

Oral administration of a single dose of ondansetron did not result in a reduction in intravenous rehydration use. In children without dehydration, ondansetron does not improve clinical outcomes.

**SEE EDITORIAL,** **P. 266.**

## Introduction

### Background

Diarrhea accounts for greater than 500,000 deaths annually in children younger than 5 years.^1^, ^2^ A critical advance has been the introduction of oral rehydration therapy employing oral rehydration solution. However, oral rehydration solution use has stagnated in most countries.^2^ When children in settings with limited health care services have fluid losses that cannot be replaced orally because of intractable vomiting, death is a potential outcome. Vomiting significantly affects oral rehydration therapy success,^3^ and in Karachi, Pakistan, 80% of children who develop severe dehydration have persistent vomiting, with a high frequency in the first 6 hours of therapy.^4^ Consequently, antiemetic administration and antibiotic use are widespread,^5^ along with intravenous fluid administration.

**Editor’s Capsule Summary**

*What is already known on this topic*

In high-income countries, ondansetron can reduce intravenous fluid use.

*What question this study addressed*

In a middle-income country, does oral administration of a single dose of oral ondansetron reduce the rate of intravenous fluid administration in children with vomiting and diarrhea but without dehydration?

*What this study adds to our knowledge*

In this trial of 626 children, a single dose of oral ondansetron did not reduce intravenous fluid use. No serious adverse events were reported.

*How this is relevant to clinical practice*

Clinicians should not prescribe ondansetron to vomiting children without evidence of dehydration.

### Importance

Oral rehydration therapy failures occur primarily because of persistent vomiting and an inability to drink.^6^ Consequently, World Health Organization (WHO) protocols recommend intravenous fluid therapy administration in such circumstances, which may divert resources from more needy patients. An easy-to-administer, effective, safe, and affordable antiemetic agent has the potential to save lives. WHO also recommends that antiemetics not be given to young children with diarrhea or dysentery.^7^ Although older antiemetic agents had unacceptable adverse effects and limited efficacy,^8^ recent research has demonstrated that a single ondansetron dose can reduce vomiting, intravenous rehydration use, and hospitalization while improving oral rehydration therapy efficacy.^9^, ^10^, ^11^

### Goals of This Investigation

Ondansetron use in low- and middle-income countries has been evaluated only in small, single-center trials,^12^, ^13^, ^14^ and none were designed to answer questions in regard to intravenous rehydration. Therefore, there remains a need for definitive evidence to guide ondansetron usage. We hypothesized that, compared with placebo, single-dose oral ondansetron administration to children in 2 emergency departments (EDs) in Pakistan who had vomiting as a result of acute gastroenteritis would reduce the probability of receiving intravenous fluid rehydration. In this study, we included only children without evidence of dehydration^15^ because we wanted to focus on and be able to analyze separately a low-risk population.

## Materials and Methods

### Study Design and Setting

Between July 3, 2014, and January 12, 2017, we conducted a double-blind, placebo-controlled, randomized superiority trial in the EDs of 2 International Organization for Standardization–certified Aga Khan institutions in Pakistan; the University Hospital, Karachi; and the Aga Khan Hospital for Women and Children, Kharadar, Karachi. The Aga Khan University Hospital is a 500-bed hospital, and the ED provides care to approximately 20,000 patients annually. It is the only ED with dedicated pediatric emergency medicine faculty providing direct patient care. The Aga Khan Hospital for Women and Children is a 48-bed hospital for women and children and includes an ED that is open 24 hours per day, 7 days per week.

The protocol was approved by the Conjoint Health Research Ethics Board of the University of Calgary and the Ethics Review Committee of Aga Khan University, Pakistan. The trial was reviewed and published as a summary by *The Lancet* (protocol 14PRT/2519). Registration was performed before the enrollment of any participants.

### Selection of Participants

Eligible participants were aged 0.5 to 5.0 years and had symptoms consistent with gastroenteritis, defined by the presence of greater than or equal to 1 episode of vomiting within the 4 hours preceding triage and greater than or equal to 1 episode of diarrhea during the illness.^16^ The minimum age is in keeping with that of previous trials,^16^ the age at which the smallest study dose (2 mg) could be administered, and reflects an age below which alternative diagnoses play a more prominent role. The requirement for recent emesis was selected to identify children who would benefit from ondansetron administration during the encounter. Eligible children had no evidence of dehydration assessed with the WHO dehydration tool.^7^ This population was important to study because we believed they were at lower risk of potential complications associated with ondansetron administration compared with children who are dehydrated because the latter group is more likely to have electrolyte abnormalities and perhaps more likely to receive intravenous rehydration without a proper trial of oral rehydration therapy. As such, we did not want to combine the 2 groups of children into a single trial, and thus we are currently conducting a clinical trial evaluating the use of ondansetron in children with evidence of dehydration in the same milieu (ClinicalTrials.gov NCT01870648).

We additionally excluded children with any of the following: bilious or bloody vomiting, hypotension (systolic blood pressure <70 mm Hg in infants aged 1 to 12 months, <70 mm Hg+[2×age in years] in children aged 1 to 10 years, <90 mm Hg in children ≥10 years^17^), weight less than 8 kg, vomiting or diarrhea for greater than 7 days, previous abdominal surgery, known hypersensitivity to ondansetron or any serotonin-receptor antagonist, personal or family history of prolonged QT syndrome, receiving a medication listed as causing torsades de pointes (http://www.azcert.org/medical-pros/drug-lists/list-01.cfm?sort=Generic_name), previously enrolled, and those for whom follow-up was not possible. Children with malnutrition (weight for height below –3z scores of the median WHO growth standards^18^) were excluded because of their increased risk of having electrolyte abnormalities.^19^ A medical officer obtained written informed consent from a parent or guardian.

Eligible participants were randomized in a 1:1 ratio to receive a single, weight-based dose of ondansetron (GlaxoSmithKline, Philadelphia, PA), provided as an oral disintegrating tablet or placebo (provided in-kind by GlaxoSmithKline). Patients weighing 8 to 15 kg received a 2-mg dose, and those weighing greater than 15 kg received 4 mg.^16^ We did not use a milligrams per kilogram dosing regimen because within the dose range of 0.13 to 0.26 mg/kg, higher doses of ondansetron do not appear to be superior to lower ones, nor are they associated with increased adverse effects.^20^ The oral disintegrating tablet was placed on the top of the tongue or along the inner aspect of the buccal mucosa, and the child was instructed to swallow 5 seconds later.^16^ Uncooperative children were assisted by the medical officer until they swallowed. Oral rehydration therapy began 15 minutes later. Children who vomited within 15 minutes of medication administration received a second dose.^16^ The active and placebo oral disintegrating tablets were identical in size, appearance, taste, and smell.^16^ The placebo contained the inactive ingredients of the active drug: aspartame, gelatin, mannitol, methylparaben sodium, propylparaben sodium, and strawberry flavor.

Randomization was stratified by study center and age (<18 and ≥18 months), using variable block sizes of 4 to 6. Allocation was concealed through the use of an Internet-based randomization service (http://www.randomize.net). A prespecified computer-generated randomization list with associated kit numbers was sent directly to the University of Calgary research pharmacist from http://www.randomize.net through password-protected files. At patient enrollment, http://www.randomize.net randomly assigned treatment and then randomly selected a kit number containing the assigned treatment.

After randomization, the medical officer retrieved the assigned randomization kit, which contained 2 oral disintegrating tablets (ie, one extra dose in case of vomiting). The drugs kits were prepared, packed, and shipped to study sites in identical containers by the Research Pharmacy–Alberta Health Services, Calgary Zone, according to the randomization list. Patients, treating physicians, investigators, and data assessors were masked to treatment allocation.

### Interventions

After the experimental intervention was provided, participants received therapy according to WHO standards of care and as deemed necessary by the emergency physician. The protocol reinforced the importance of oral rehydration solution use for the first 4 hours while the child was monitored and the caregiver was taught how to prepare and administer oral rehydration solution. The volume administered was based on the child’s weight,^15^ and caregivers were educated on administering a teaspoonful every 1 to 2 minutes (<2 years) or instructing the child to sip from a cup (≥2 years), as appropriate. Breastfeeding mothers continued whenever the child wanted. Children were monitored for evidence of deterioration. Caregivers resumed rehydration 10 minutes after each vomiting episode. Caregivers were provided with a 2-day oral rehydration solution supply at discharge.

After discharge, caregivers were instructed to administer as much fluid as possible to prevent dehydration and to continue feeding. Exclusively breastfed children were administered oral rehydration solution in addition to breast milk. An additional 100 mL of oral rehydration solution was to be provided for every loose stool.^7^ Participants received one zinc tablet (20 mg daily) for 14 days.

### Methods of Measurement

While patients were in the hospital, study-funded medical officers documented the volumes of oral and intravenous fluids administered, and the frequency and volumes of vomiting, diarrhea, and urination. Because separating urine output from stool is challenging in young children with diarrhea, urine collection bags were provided for children who were not capable of urinating into measurement containers. Dehydration parameters and vital signs were documented at the 4-hour assessment. Dehydration severity was assessed with the WHO severity assessment algorithm, which classifies children as having “some” dehydration if 2 or more of the following are present: restlessness or irritability, sunken eyes, drinking eagerly or thirsty, and skin recovers slowly from pinching.^7^

Caregivers used a diary in which they recorded vomiting and diarrhea episodes at home. Discharged patients were reassessed 24-hours after discharge at their home or the enrolling institution. At the revisit, clinical nurses determined whether the child required a further ED visit, had received intravenous fluid treatment, or had been hospitalized. If there were no signs of dehydration and symptoms had subsided, 48- and 72-hour follow-up was by telephone. For patients with ongoing symptoms or evidence of dehydration, an in-person 24-hour reassessment was required. In case of noncompliance, caregivers were contacted by telephone and information was gathered in regard to the child’s health and dehydration severity daily for 7 days. Participating hospital records were reviewed to confirm caregivers’ reports. If the child was admitted or received treatment at a different institution, information was collected from caregivers by telephone.

### Outcome Measures

The primary outcome was intravenous rehydration, defined as the administration of an isotonic fluid at greater than or equal to 20 mL/kg during 4 hours for the purpose of rehydration within 72 hours of randomization. This pragmatic outcome allowed the occurrence of the primary outcome in children who received maintenance plus replacement of losses and those who received a fluid bolus but did not include those who only received antibiotics or maintenance fluids (eg, 4 mL/kg per hour for those weighing <10 kg). The 72-hour time frame was selected to enable a balancing of the potential short-term benefits of the intervention (ie, reduction in intravenous line insertion at the index visit) with the potential adverse effects (ie, increased diarrhea and revisits).

Secondary endpoints were the presence and frequency of vomiting during the 4-hour observation period; hospitalization for greater than 24 hours, defined as ED arrival until hospital discharge; volume of oral rehydration solution consumed (milliliters/kilogram) during the 4-hour observation period; presence of some dehydration during the 72-hour follow-up period among discharged children; number of diarrheal stools during the 72-hour follow-up period; and treatment failure. The latter composite measure included any of the following: intravenous rehydration as defined above, nasogastric rehydration for greater than 24 hours, or death within 72 hours.

Exploratory outcomes included serious adverse events, semi-ICU (for children in shock but not requiring mechanical ventilation) and ICU admissions, volume of diarrhea during the 4-hour study period, and 72-hour hydration status. Parental report was used to identify delayed serious adverse events.

### Primary Data Analysis

The analysis plan for this superiority trial was prespecified and performed masked to treatment allocation. The probability that a control arm study participant would experience the primary outcome measure was estimated according to data reported by the International Study Group on Reduced-Osmolarity ORS Solutions (17%).^21^ In accordance with the former study, investigators with extensive experience working in the participating EDs determined that a point estimate of 20% should be used for the control group. According to the opinion of experts in the field and our team members who work at the study sites, the maximum number of patients meeting study eligibility criteria who should be treated with oral ondansetron to prevent one failure is 10 (ie, minimum clinically meaningful difference of 10 percentage points). We anticipated a 5% loss to follow-up.^22^ Using a 2-sided type I error of 0.05 and 90% power, the required sample size was 602 participants. Because of delays in data entry and data completeness concerns, the steering committee requested the recruitment of an additional 25 patients. All randomized children were included in the analyses, which followed the intention-to-treat principle.

Patient characteristics are presented as frequencies and percentages for categorical data and medians (interquartile range [IQR]) for continuous data. The proportion of children receiving intravenous rehydration by 72 hours was analyzed by comparing proportions with a Mantel-Haenszel test, stratified by clinical center. Secondary analyses of the primary outcome used a logistic regression model fitted with treatment group and a priori identified baseline covariates (antiemetic, antibiotics, zinc administration prerandomization, and number of vomiting episodes in the 24 hours before enrollment) that potentially were associated with the outcome. We conducted prespecified subgroup analyses based on subject age (ie, <2 years, 2 to 5 years) and presence of the WHO definition of diarrhea (ie, ≥3 episodes in a 24-hour period). A post hoc analysis reanalyzed the primary outcome, including as an event only patients who experienced the primary endpoint and developed severe dehydration.

The secondary outcomes of vomiting, hospitalization greater than 24 hours, development of some dehydration by 72 hours, and treatment failure were analyzed with a Mantel-Haenszel test stratified by clinical center. The median differences of the continuous variables vomiting frequency, volume of oral rehydration solution consumed, and diarrheal stool frequency were computed with the Hodges-Lehmann estimator, whereas statistical significance was assessed with a Van Elteren test stratified by clinical center.

Every effort was made to identify and retrieve complete data. Missing data were not imputed. A 2-tailed *P*<.05 was considered to be statistically significant for the primary outcome, and a Bonferroni corrected threshold of 0.007 was used for the 7 secondary outcomes. The analysis was performed with SPSS (version 22.0; SPSS, Inc., Chicago, IL) and SAS (version 9.4; SAS Institute, Inc., Cary, NC).

## Results

### Characteristics of Study Subjects

**Figure 1:**
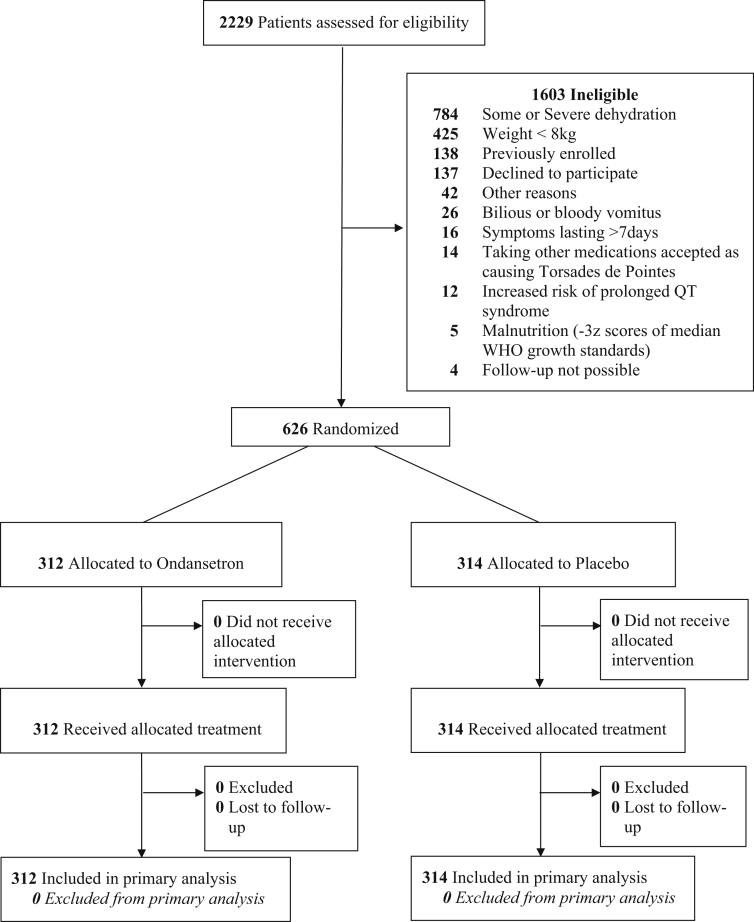
Trial profile.

A total of 2,229 patients were assessed for inclusion and 626 were randomized (Figure 1). Baseline characteristics were similar in the 2 groups (Table 1). The intervention or placebo medication administered was vomited by 7 (2.2%) and 6 (1.9%) children in the ondansetron and placebo groups, respectively.

**Table 1:** Baseline clinical characteristics of participants by treatment group.

	Ondansetron (n=312)	Placebo (n=314)
Age, mo	15 (10–25)	16 (11–27)
Male	189 (60.6)	183 (58.3)
Weight, kg	9.7 (8.5–12.0)	10.0 (8.5–11.8)
Chronic medical conditions	1 (0.3)	4 (1.3)
Interval of last vomiting episode to medication administration, h	1.58 (0.84–2.90)	1.67 (0.92–2.92)
Maximal vomiting episodes per 24-h period	4 (3–6)	4 (3–6)
Vomiting episodes past 24 h	4 (2–6)	4 (2–5)
Vomiting duration, days	1 (1–2)	1 (1–2)
Maximal diarrheal episodes per 24-h period	4 (2–6)	3 (2–6)
Diarrheal episodes past 24 h	3 (1–5)	2 (2–4)
Diarrhea duration, days	1 (1–2)	1 (1–2)
Fever	133 (42.6)	113 (36.0)
Previous ED visit, current illness	41 (13.1)	38 (12.1)
Previous intravenous rehydration, current illness	17 (5.4)	13 (4.2)
Previous hospitalization, current illness	3 (1.0)	2 (0.6)
Rotavirus vaccine receive	113 (36.2)	114 (36.3)
Exclusively breastfed	24 (7.7)	19 (6.1)
Triage pulse rate, beats/min	133 (126–146)	132 (122–145)
Triage respiratory rate, breaths/min	30 (26–32)	30 (26–32)
Triage capillary refill ≥2 s	5 (1.6)	6 (1.9)
Triage temperature (°C/°F), rectal adjusted	37.7 (37.5–38.1)/99.9 (99.5-100.6)	37.7 (37.5–38.1)/99.9 (99.5-100.6)
Clinical Dehydration Scale score^33^	0 (0–1)	0 (0–1)

Data are presented as No. (%) or median (IQR). Fever was defined as an adjusted rectal temperature of greater than or equal to 38.0°C (100.4°F). Axillary and oral temperatures were adjusted to rectal temperatures by adding 1.1°C and 0.6°C, respectively.^34^ Some children had received more than one medication in the past 24 hours.

### Main Results

**Figure 2:**
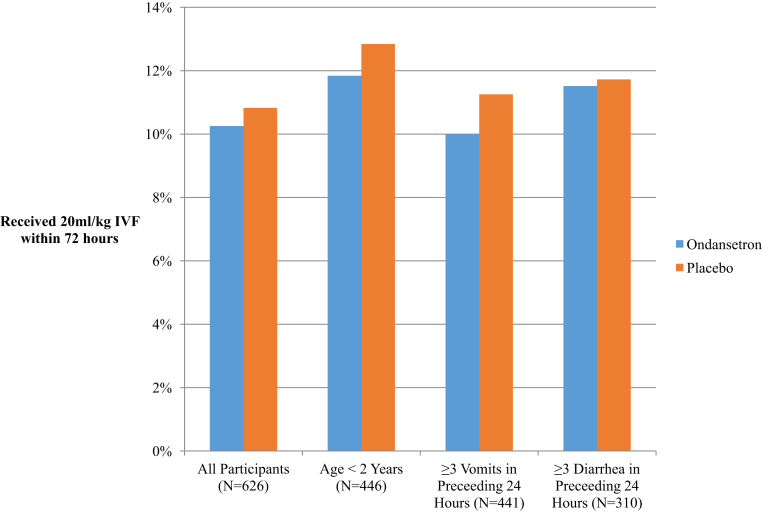
Subgroup analyses. *IVF*, Intravenous fluids.

Overall, 12.0% of children (75/626) received intravenous fluids during the study period (placebo 38/314 [12.1%]; ondansetron 37/312 [11.9%]; odds ratio [OR] 0.98; 95% confidence interval [CI] 0.60 to 1.61; difference 0.2%; 95% CI of the difference –4.9% to 5.4%). Administration of an isotonic intravenous rehydration solution at greater than or equal to 20 mL/kg during 4 hours for the purpose of rehydration within 72 hours of randomization was 10.8% (34/314) in the placebo group and 10.3% (32/312) in the ondansetron group (OR 0.95; 95% CI 0.56 to 1.59) (Table 2). Secondary analysis using a logistic regression model fitted with treatment group and adjusted for covariates yielded an OR=0.94 (95% CI 0.56 to 1.57). Administration of other antiemetic agents prerandomization was not associated with the outcome (OR 1.41; 95% CI 0.71 to 2.77) (Table 3). Inclusion in the regression model of interaction terms including treatment group and age, presence of greater than or equal to 3 diarrheal stools in the preceding 24 hours, and presence of greater than or equal to 3 vomiting episodes in the preceding 24 hours did not significantly alter the results (Table 4). Subgroup analyses are presented in Figure 2. No study participant developed severe dehydration.

**Table 2:** Participant clinical outcomes by treatment group.

	Ondansetron, N=312	Placebo, N=314	% Difference (95% CI)	Median Difference (95% CI)*
**Primary outcome**				
Intravenous fluids administered, ≥20 mL/kg	32 (10.3)	34 (10.8)	–0.6 (–5.5 to 4.3)	
**Secondary outcomes, time 0–4 h**				
Vomiting, yes	61 (19.6)	75 (24.0)	–4.3 (–10.8 to 2.1)	
Vomiting, frequency	0 (0 to 0)	0 (0 to 0)		0 (0 to 0)
Volume of oral fluids, mL/kg per hour	3.4 (1.9 to 5.7)	3.2 (1.9 to 5.9)		0.2 (–0.4 to 0.4)
**Secondary outcomes, time >4 h**				
Hospitalization >24 h	12 (3.8)	11 (3.5)	0.3 (–2.8 to 3.5)	
Presence of some dehydration within 72 h	18 (5.8)	24 (7.6)	–1.9 (–5.9 to 2.1)	
Diarrhea, frequency during 72 h postenrollment	4 (1 to 7)	4 (1 to 6)		0 (–1 to 0)
Treatment failure	32 (10.3)	34 (10.8)	–0.6 (–5.5 to 4.3)	
**Other outcomes**				
4-h pulse rate, beats/min	128 (120 to 138)	128 (120 to 136)		0 (–2 to 2)
4-h respiratory rate, breaths/min	30 (26 to 32)	28 (25 to 32)		0 (–2 to 0)
4-h capillary refill ≥2 s	3 (1.0)	2 (0.6)	0.3 (–1.5 to 2.2)	
**4-h WHO Dehydration Severity Score**				
None	305 (97.8)	305 (97.1)	0.6 (–2.1 to 3.4)	
Some	7 (2.2)	9 (2.9)	–0.6 (–3.4 to 2.1)	
Severe	0	0		
4-h Clinical Dehydration Scale score^33^	0 (0 to 0)	0 (0 to 0)		0 (0 to 0)
4-h diarrhea, volume, mL/kg per hour	2.5 (0.0 to 5.0)	2.5 (0.0 to 2.5)		0 (0 to 0)
4-h vomiting, volume, mL/kg per hour	0 (0 to 0)	0 (0 to 0)		0 (0 to 0)
4-h urine output, volume, mL/kg per hour	0.79 (0.44 to 1.78)	0.63 (0.30 to 1.61)		–0.01 (–0.20 to 0)
**4-h disposition**				
Discharge	292 (93.6)	288 (91.7)	1.9 (–2.3 to 6.1)	
Ongoing observation/oral rehydration	9 (2.9)	11 (3.5)	–0.6 (–3.6 to 2.3)	
Intravenous rehydration	11 (3.5)	15 (4.8)	–1.3 (–4.6 to 2.0)	

Discrete variables are provided as No. (%) and continuous variables are presented as median (IQR). Hospital length of stay was defined as a total length of stay from ED arrival until discharge. Dehydration status was assessed with the WHO dehydration assessment approach.^15^ Diarrhea was defined as loose or liquid stools.^35^ Treatment failure was a composite outcome measure defined by the occurrence of any of the following: intravenous rehydration (≥20 mL/kg during 4 hours); nasogastric rehydration for greater than 24 hours; and death within 72 hours from any cause, in or out of hospital.

**Table 3:** Medication administration pre-ED, in the ED, and post-ED, by treatment group.

	Ondansetron (n=312), No. (%)	Placebo (n=314), No. (%)
**Medications administered, past 24 h**	90 (28.8)	102 (32.5)
**Antacids**		
Omeprazole/ranitidine	2 (0.6)	5 (1.6)
**Antipyretics**	32 (10.3)	24 (7.6)
Acetaminophen	26 (8.3)	24 (7.6)
Ibuprofen	6 (1.9)	0
**Antibiotics/anthelmintics**	31 (9.9)	44 (14.0)
Azithromycin/clarithromycin	4 (1.3)	4 (1.3)
Cefixime/cefotaxime/ceftriaxone	10 (3.2)	12 (3.8)
Diloxanide/mebendazole	2 (0.6)	8 (2.5)
Metronidazole	12 (3.8)	25 (8.0)
Other	8 (2.6)	4 (1.3)
**Any antiemetics**	48 (15.4)	55 (17.5)
Dimenhydrinate	16 (5.1)	16 (5.1)
Domperidone	33 (10.6)	42 (13.4)
Metoclopramide	1 (0.3)	0
**Antihistamines/anticholinergics**		
**Cetirizine/clemastine/cyclizine/diphenhydramine**	4 (1.3)	6 (1.9)
**Nutrition**		
Zinc	9 (2.9)	12 (3.8)
**Probiotics**		
*Saccharomyces boulardii*	4 (1.3)	9 (2.9)
**ED cointerventions**		
**Antacid**	10 (3.2)	3 (1.0)
Omeprazole	8 (2.6)	3 (1.0)
Ranitidine	2 (0.6)	0
**Antibiotic**	56 (18.0)	67 (21.3)
Administered in ED prerandomization	2 (0.6)	3 (1.0)
Albendazole	1 (0.3)	0
Amoxicillin	0	1 (0.3)
Azithromycin	8 (2.6)	6 (1.9)
Cefixime	11 (3.5)	7 (2.2)
Ceftriaxone	16 (5.1)	27 (8.6)
Ciprofloxacin	4 (1.3)	7 (2.2)
Metronidazole	16 (5.1)	19 (6.1)
**Antiemetic**		
Administered in ED prerandomization	2 (0.6)	2 (0.6)
Dimenhydrinate	51 (16.3)	59 (18.8)
Domperidone	2 (0.6)	1 (0.3)
Ondansetron	43 (13.8)	52 (16.6)
Antiemetic	6 (1.9)	5 (1.6)
**Antihistamine**		
Cetirizine	2 (0.6)	2 (0.6)
**Antipyretic**	44 (14.1)	36 (11.5)
Acetaminophen	33 (10.6)	26 (8.3)
Ibuprofen	11 (3.5)	10 (3.2)
**Other**		
Zinc administered in ED prerandomization	3 (1.0)	1 (0.3)
Zinc	52 (16.7)	59 (18.8)
*S boulardii*	89 (28.5)	97 (30.9)
Antibiotic prescribed at/after discharge	53 (17.0)	60 (19.1)
Any antibiotics during the study period	56 (17.9)	67 (21.3)

Fever was defined as an adjusted rectal temperature of greater than or equal to 38.0°C (100.4°F). Axillary and oral temperatures were adjusted to rectal temperatures by adding 1.1°C and 0.6°C, respectively.^34^ Some children had received more than one medication in the past 24 hours.

**Table 4:** Multiple regression model of the primary outcome (intravenous fluids administration, ≥20 mL/kg, within 72 hours of randomization), including a priori–identified interaction terms.

Variable	OR (95% CI)
**Antiemetic in preceding 24 h**	
Yes	1.58 (0.79–3.15)
No	[Reference]
**Zinc in preceding 24 h**	
Yes	0.35 (0.04–2.70)
No	[Reference]
**Antibiotic in preceding 24 h**	
Yes	0.90 (0.38–2.11)
No	[Reference]
**≥3 vomiting episodes in preceding 24 h**	
Yes	1.13 (0.49–2.62)
No	[Reference]
**≥3 diarrhea episodes in preceding 24 h**	
Yes	1.22 (0.59–2.52)
No	[Reference]
Age, mo	0.98 (0.95–1.02)
**Interaction terms**	
Treatment group×no. vomiting episodes in preceding 24 h	0.70 (0.22–2.22)
Treatment group×no. of diarrheal episodes in preceding 24 h	0.99 (0.35–2.85)
Treatment group×age, mo	0.99 (0.95–1.04)

Model characteristics included the following: omnibus test comparing the fitted model against the intercept-only model, *P*=.76; Nagelkerke *R*^2^=0.022.

Overall, 24.0% of children (75/314) in the placebo group vomited during the 4-hour study observation period compared with 19.6% (61/312) in the ondansetron group (OR 0.77; 95% CI 0.53 to 1.13). There were no differences in the median number of vomiting episodes (ondansetron 0, IQR 0 to 0; placebo 0, IQR 0 to 0) or in the volume of oral fluids consumed (milliliters/kilogram per hour) (ondansetron 3.4, IQR 1.9 to 5.7; placebo 3.2, IQR 1.9 to 5.9) during the 4-hour study observation period. The proportions of children hospitalized greater than 24 hours (OR 1.11; 95% CI 0.48 to 2.55) and who developed some dehydration by 72 hours (OR 0.74; 95% CI 0.39 to 1.39) did not differ between groups. The number of diarrheal stools during the 72-hour follow-up period was similar between groups (ondansetron 4, IQR 1 to 7; placebo 4, IQR 1 to 6). Because no children died and none received nasogastric rehydration, the composite outcome of treatment failure was equal to that of intravenous rehydration. Among patients who had laboratory testing performed, there were no significant differences (Table 5).

**Table 5:** Baseline blood tests.

	Ondansetron (N=312)		Placebo (N=314)		Mean Difference (95% CI)
	No. (%)	Mean (SD)	No. (%)	Mean (SD)	
Bicarbonate, mmol/L	43 (13.8)	17.3 (4.1)	43 (13.7)	16.6 (3.6)	0.7 (–1.0 to 2.4)
Sodium, mmol/L	42 (13.5)	137.8 (3.8)	45 (14.3)	138.0 (4.0)	–0.2 (–1.9 to 1.4)
Potassium, mmol/L	43 (13.8)	4.3 (0.5)	45 (14.3)	4.2 (0.5)	0.08 (–0.1 to 0.3)
Chloride, mmol/L	42 (13.5)	104.1 (5.5)	42 (13.4)	103.4 (4.8)	0.6 (–1.6 to 2.9)
Urea nitrogen, mmol/L	6 (1.9)	15.8 (3.0)	6 (1.9)	9.5 (4.2)	6.3 (1.7 to 11.0)
Creatinine, μmol/L	11 (3.5)	0.44 (0.23)	8 (2.5)	0.34 (0.11)	0.10 (–0.08 to 0.28)
WBC count, ×10^9^/L	43 (13.8)	13.5 (6.5)	54 (17.2)	11.8 (4.5)	1.8 (–0.4 to 4.0)
Platelets, ×10^9^/L	43 (13.8)	411 (127)	54 (17.2)	412 (156)	–1 (–60 to 57)
Hemoglobin, g/L	43 (13.8)	11.3 (1.4)	54 (17.2)	11.2 (1.4)	0.1 (–0.5 to 0.7)

We recorded no serious adverse events or admissions to the semi-ICU or ICU. Six adverse events were reported in each study group. No important differences were noted between groups for type or severity of event. The median volume of diarrhea during the 4-hour study observation period was similar between groups (ondansetron 2.5 mL/kg per hour, IQR 0 to 5.0; placebo 2.5 mL/kg per hour, IQR 0 to 2.5). Last, groups were similar with respect to the proportion of children who had some dehydration at 72 hours (ondansetron 18/312 [5.8%]; placebo 24/314 [7.6%]; difference 1.9%; 95% CI of the difference –2.1% to 5.9%).

## Limitations

Our study had several limitations. The observed event rate was lower than anticipated in the control group (12.1% versus 20%). As a result, the variance of the observed rates was lower and the power to detect a 10-percentage-point difference higher. Therefore, we do not believe this alters our interpretation of our findings, and it highlights the need to target ondansetron use for children with evidence of dehydration, failure of oral rehydration therapy, or recent and significant vomiting.^23^ Moreover, despite significant increases in ondansetron use in the United States during the past decade, intravenous rehydration rates have not changed significantly.^24^ Taken together, these findings highlight the importance of focusing ondansetron use for children at greatest risk of oral rehydration therapy failure.

We had intended to collect and perform testing on stool specimens to determine whether the response varied by pathogen. However, testing was performed in only 5% of the sample, and thus valid analyses could not be performed. In addition, many study participants were coadministered other medications, including antibiotic and antiemetic agents including domperidone. Although this was adjusted for in the analysis and previous studies have not found it to be an effective antiemetic agent,^25^ its coadministration may have played a role in minimizing any effect ondansetron may have had.

## Discussion

Oral administration of a single dose of ondansetron did not reduce the proportion of children administered intravenous fluid rehydration in our trial. Analysis of secondary outcomes, including presence and frequency of vomiting, hospitalization rate, oral rehydration fluid volume consumed, development of dehydration, and diarrheal stool frequency, showed no evidence of benefit attributable to ondansetron administration. Accounting for potential risk factors for oral rehydration therapy failure and intravenous rehydration did not significantly alter the findings.

We specifically excluded children with dehydration because we wanted to focus on a low-risk population; consequently, the children in our study, compared with those in previous studies,^26^ were less unwell. Our findings similarly differ. A meta-analysis of 3 trials recently reported a reduction in intravenous rehydration use in patients treated with ondansetron compared with placebo.^27^ However, all 3 trials required the presence of dehydration. Specifically, the 2 studies^16^, ^28^ performed in the United States required the presence of mild to moderate dehydration. The third study, performed in India, required the presence of some dehydration, but it was the only one to not report a reduction in intravenous rehydration rates.^12^ We enrolled children without dehydration according to local expert opinion that children without dehydration might demonstrate symptom progression and develop dehydration. Thus, through oral rehydration therapy promotion, early ondansetron administration was hypothesized to be capable of preventing intravenous rehydration use. Our findings thus put into context the clinical features of children unlikely to benefit from ondansetron administration.

Another key difference from previous studies relates to the frequency of vomiting. Our eligibility criteria required greater than or equal to 1 episode of vomiting during the 4 hours preceding triage. Although this criterion has previously been used,^16^ the frequency of vomiting in that study exceeded 9 episodes in the preceding 24 hours, whereas participants in our study reported just 4 episodes of vomiting in the preceding 24 hours. Although Roslund et al^28^ did not specify a minimum frequency of vomiting episodes, study participants had a median of 10 episodes before presentation, with a median duration of vomiting of 1 day in the ondansetron group. Danewa et al^12^ required a minimum of 2 episodes of vomiting within the last 6 hours, and the cohort had a median of 6 episodes. Although our analysis stratified by frequency of vomiting did not reveal a significant difference between groups, the low number of vomiting episodes among participants preceding enrollment and the low proportion of control participants who vomited (24%) during the study, compared with results of previous studies (≈35%),^16^ may further explain the lack of beneficial effect identified.

As noted, intravenous rehydration use was lower in our study than we had anticipated. Although that may have been due to the aforementioned clinical features (ie, absence of dehydration and fewer vomiting episodes), ondansetron administration was not our only intervention. We also ensured that caregivers received education on the provision of oral rehydration solution, using appropriate oral rehydration therapy techniques, and they were provided with zinc therapy, as is recommended by WHO.^29^, ^30^ These adjunctive therapies may have played a role in reducing the frequency of intravenous rehydration use.

Our findings highlight the adjunctive, and usually not indicated, use of additional therapies in this population. In addition to the administration of ondansetron to 50% of our study population (as per study protocol), 17% of study participants had received an antiemetic agent before ED arrival and an additional 36% were administered an antiemetic agent after enrollment (ie, outside of study protocol). In the ondansetron study arm, 43 children (14%) received a second dose of ondansetron while in the ED despite the responsible physician’s being aware that they had a 50% chance of already having received a dose of ondansetron as part of the study protocol. Thus, there is a clear belief that ondansetron administration is beneficial when administered to children with mild disease.

We also found antibiotic use to be surprisingly common both before ED arrival and in the ED itself despite that international guidelines recommend against antibiotic treatment for nonbloody diarrhea.^31^ Our findings are in keeping with those of other reports, including the recently completed Malnutrition and Enteric Disease birth cohort study (2,134 children from 8 sites in Bangladesh, Brazil, India, Nepal, Pakistan, Peru, South Africa, and the United Republic of Tanzania) that reported antibiotic use in 44% of cases of nonbloody diarrhea.^32^

In summary, our findings do not provide evidence to support the routine administration of a single dose of oral ondansetron for the prevention of intravenous fluid administration in children with gastroenteritis but without evidence of dehydration.

## References

[1] Liu L., Oza S., Hogan D. (2016). Global, regional, and national causes of under-5 mortality in 2000-15: an updated systematic analysis with implications for the Sustainable Development Goals. Lancet.

[2] Santosham M., Chandran A., Fitzwater S. (2010). Progress and barriers for the control of diarrhoeal disease. Lancet.

[3] Kamala C.S., Vishwanathakumar H.M., Shetti P.M. (1996). Management of diarrhea in a DTU. Indian Pediatr.

[4] Ibrahim S., Isani Z. (1997). Sagodana based verses rice based oral rehydration solution in the management of acute diarrhoea in Pakistani children. J Pak Med Assoc.

[5] Fisher Walker C.L., Taneja S., Lamberti L.M. (2016). Management of childhood diarrhea among private providers in Uttar Pradesh, India. J Glob Health.

[6] Bahl L., Sharma V.K., Kaushal R.K. (1997). Experience with diarrhea training and treatment unit in Shimla. Indian Pediatr.

[7] World Health Organization (2013). Pocket Book of Hospital Care for Children: Guidelines for the Management of Common Childhood Illnesses.

[8] Freedman S.B., Fuchs S. (2004). Antiemetic therapy in pediatric emergency departments. Pediatr Emerg Care.

[9] Carter B., Fedorowicz Z. (2012). Antiemetic treatment for acute gastroenteritis in children: an updated Cochrane systematic review with meta-analysis and mixed treatment comparison in a Bayesian framework. BMJ Open.

[10] Freedman S.B., Pasichnyk D., Black K.J. (2015). Gastroenteritis therapies in developed countries: systematic review and meta-analysis. PLoS One.

[11] Freedman S.B., Ali S., Oleszczuk M. (2013). Treatment of acute gastroenteritis in children: an overview of systematic reviews of interventions commonly used in developed countries. Evid Based Child Health.

[12] Danewa A.S., Shah D., Batra P. (2016). Oral ondansetron in management of dehydrating diarrhea with vomiting in children aged 3 months to 5 years: a randomized controlled trial. J Pediatr.

[13] Golshekan K., Badeli H., Rezaieian S. (2013). Effect of oral ondansetron on decreasing the vomiting associated with acute gastroenteritis in Iranian children. Iran J Pediatr.

[14] Rerksuppaphol S., Rerksuppaphol L. (2013). Randomized study of ondansetron versus domperidone in the treatment of children with acute gastroenteritis. J Clin Med Res.

[15] World Health Organization (2005). Pocket Book of Hospital Care for Children.

[16] Freedman S.B., Adler M., Seshadri R. (2006). Oral ondansetron for gastroenteritis in a pediatric emergency department. N Engl J Med.

[17] Kleinman M.E., Chameides L., Schexnayder S.M. (2010). Part 14: pediatric advanced life support: 2010 American Heart Association guidelines for cardiopulmonary resuscitation and emergency cardiovascular care. Circulation.

[18] World Health Organization; United Nations Children's Fund (2009). WHO Child Growth Standards and the Identification of Severe Acute Malnutrition in Infants and Older Children.

[19] Freedman S.B., Uleryk E., Rumantir M. (2014). Ondansetron and the risk of cardiac arrhythmias: a systematic review and postmarketing analysis. Ann Emerg Med.

[20] Freedman S.B., Powell E.C., Nava-Ocampo A.A. (2010). Ondansetron dosing in pediatric gastroenteritis: a prospective cohort, dose-response study. Paediatr Drugs.

[21] International Study Group on Reduced-Osmolarity ORS Solutions (1995). Multicentre evaluation of reduced-osmolarity oral rehydration salts solution. Lancet.

[22] Soofi S., Ahmed S., Fox M.P. (2012). Effectiveness of community case management of severe pneumonia with oral amoxicillin in children aged 2-59 months in Matiari District, rural Pakistan: a cluster-randomised controlled trial. Lancet.

[23] Cheng A. (2011). Emergency department use of oral ondansetron for acute gastroenteritis-related vomiting in infants and children. Paediatr Child Health.

[24] Freedman S.B., Hall M., Shah S.S. (2014). Impact of increasing ondansetron use on clinical outcomes in children with gastroenteritis. JAMA Pediatr.

[25] Marchetti F., Bonati M., Maestro A. (2016). Oral ondansetron versus domperidone for acute gastroenteritis in pediatric emergency departments: multicenter double blind randomized controlled trial. PLoS One.

[26] Fedorowicz Z., Jagannath V.A., Carter B. (2011). Antiemetics for reducing vomiting related to acute gastroenteritis in children and adolescents. Cochrane Database Syst Rev.

[27] Tomasik E., Ziolkowska E., Kolodziej M. (2016). Systematic review with meta-analysis: ondansetron for vomiting in children with acute gastroenteritis. Aliment Pharmacol Ther.

[28] Roslund G., Hepps T.S., McQuillen K.K. (2008). The role of oral ondansetron in children with vomiting as a result of acute gastritis/gastroenteritis who have failed oral rehydration therapy: a randomized controlled trial. Ann Emerg Med.

[29] World Health Organization Department of Child and Adolescent Health and Development/UNICEF. *Clinical Management of Acute Diarrhoea: WHO/UNICEF Joint Statement [WHO/FCH/CAH/04.7; UNICEF/PD/Diarrhoea/01]*. Geneva, Switzerland: The United Nations Children’s Fund/World Health Organization; 2004.

[30] Lazzerini M., Wanzira H. (2016). Oral zinc for treating diarrhoea in children. Cochrane Database Syst Rev.

[31] World Health Organization. *Integrated Management of Childhood Illness: Distance Learning Course, Module 4—Diarrhoea*. Geneva, Switzerland: World Health Organization; 2014.

[32] Rogawski E.T., Platts-Mills J.A., Seidman J.C. (2017). Use of antibiotics in children younger than two years in eight countries: a prospective cohort study. Bull World Health Organ.

[33] Friedman J.N., Goldman R.D., Srivastava R. (2004). Development of a clinical dehydration scale for use in children between 1 and 36 months of age. J Pediatr.

[34] Alpern E.R., Henretig F.M., Fleisher G.R., Ludwig S., Henretig F.M. (2006). Fever. Textbook of Pediatric Emergency Medicine.

[35] Freedman S.B., Powell E., Seshadri R. (2009). Predictors of outcomes in pediatric enteritis: a prospective cohort study. Pediatrics.

